# Therapeutic advances in overcoming intrauterine growth restriction induced metabolic syndrome

**DOI:** 10.3389/fped.2022.1040742

**Published:** 2023-01-11

**Authors:** Alpha Kalonda Mutamba, Xiaori He, Tao Wang

**Affiliations:** ^1^ Department of Pediatrics, Neonatology, The Second Xiangya Hospital, Central South University, Changsha, China; ^2^ Laboratory of Neonatal Disease, Institute of Pediatrics, Central South University, Changsha, China

**Keywords:** small gestational age (SGA), lipid metabolism, insulin resistance, type 2 diabetes, intrauterine growth restriction

## Abstract

Intrauterine growth restriction (IUGR) remains a great public health challenge as it affects neonatal survival and influences their normal biological development and metabolism. Several clinical researches have revealed the occurrence of metabolic syndrome, such as insulin resistance, obesity, type 2 diabetes mellitus, oxidative stress, dyslipidemia, as direct results of IUGR. Therefore, it is essential to understand its underlying mechanism, impact and develop effective therapies. The purpose of this work is to review the current knowledge on IUGR induced metabolic syndrome and relevant therapies. Here in, we elaborate on the characteristics and causes of IUGR by pointing out recent research findings. Furthermore, we discuss the impact of IUGR on different organs of the body, followed by preclinical studies on IUGR using suitable animal models. Additionally, various metabolic disorders with their genetic implications, such as insulin resistance, type 2 diabetes mellitus, dyslipidemia, obesity are detailed. Finally, the current therapeutic options used in the treatment of IUGR are summarized with some prospective therapies highlighted.

## 1. Introduction

Intrauterine growth restriction (IUGR), also called fetal growth restriction (FGR) is a condition in which the fetus is unable to develop to its full genetic and biological potential (1). This condition affects nearly 30%–50% of extremely preterm neonates (2). IUGR can be caused by naturally occurring or adverse environmental factors as revealed by studies conducted in livestock. To mimic human IUGR brought on by a variety of factors, several experimental techniques have been applied using animal models like sheep (3). Although hasty comparisons cannot be drawn between these animal models and humans, the impromptu occurrence of IUGR *via* placental insufficiency in pigs has been reported to be identical to that of humans (4, 5). IUGR causes great changes from abnormal lipid metabolism, liver inflammation to metabolic syndrome (MetS).

MetS is a constellation of morbidities such as insulin resistance, dyslipidemia, central obesity, hypertension, and glucose intolerance that includes impaired glucose tolerance or compromised fasting glycaemia and type 2 diabetes; all of which are well-documented cardiovascular disease risk factors. These metabolic abnormalities can simultaneously occur in an individual more often than expected (6). The “Developmental Origins of Health and Disease” theory evokes that a pernicious environment during early developmental periods (including fetal, infant and childhood) may lead to permanent alterations of both physiological and metabolic functions leading to adult metabolic syndrome (MetS) (7). In the principal etiology of these metabolic abnormalities, excessive fat storage in non-adipose tissues (e.g., liver) is a significant risk factor (8). The abnormal lipid metabolism in the liver has proven to be closely related to MetS (9). Except for diet and lifestyle, birth weight (BW) is considered the main relationship between abnormal hepatic accumulation of lipid and the increased incidence of MetS with its associated diseases (10).

Given the impact of IUGR on the development of MetS, there is a need to understand these conditions, review different parameters affected in IUGR models, and give an overview on current studied therapies.

## 2. Characteristics and causes of intrauterine growth restriction

IUGR is characterized as symmetrical when the weight, length, and head circumference are low compared with the standard; and asymmetrical when the brain is spared, and the head circumference is within the normal limits (11). Chromosomal syndromes (trisomy 21, 13, and 18), congenital infections (toxoplasma, other viruses, rubella, cytomegalovirus, Herpes simplex infections), dwarfisms, maternal drug use (both prescription and illegal), and some inborn metabolic errors (fatty acid oxidation disorders) can all cause symmetrical IUGR. Asymmetrical causes of IUGR are typically associated with placental insufficiency, placental dysfunction, or nutrient deficiency (12, 13).

About 13% of children born with IUGR do not “catch up” with normal growth, i.e., do not reach body height greater than −2 *z*-score (below the third centile) during the first two to four years after birth (14). Many studies have demonstrated a clear correlation between deficient *in utero* environment leading to IUGR and the occurrence of cardiometabolic disease in adulthood (15, 16). Poor gestational nutrition and low pre-pregnancy weight are the strongest predictors of IUGR, according to data from developing countries. While in developed countries, cigarette smoking is the most important single factor implicated in IUGR, followed by poor gestational nutrition (17).

**Figure 1:**
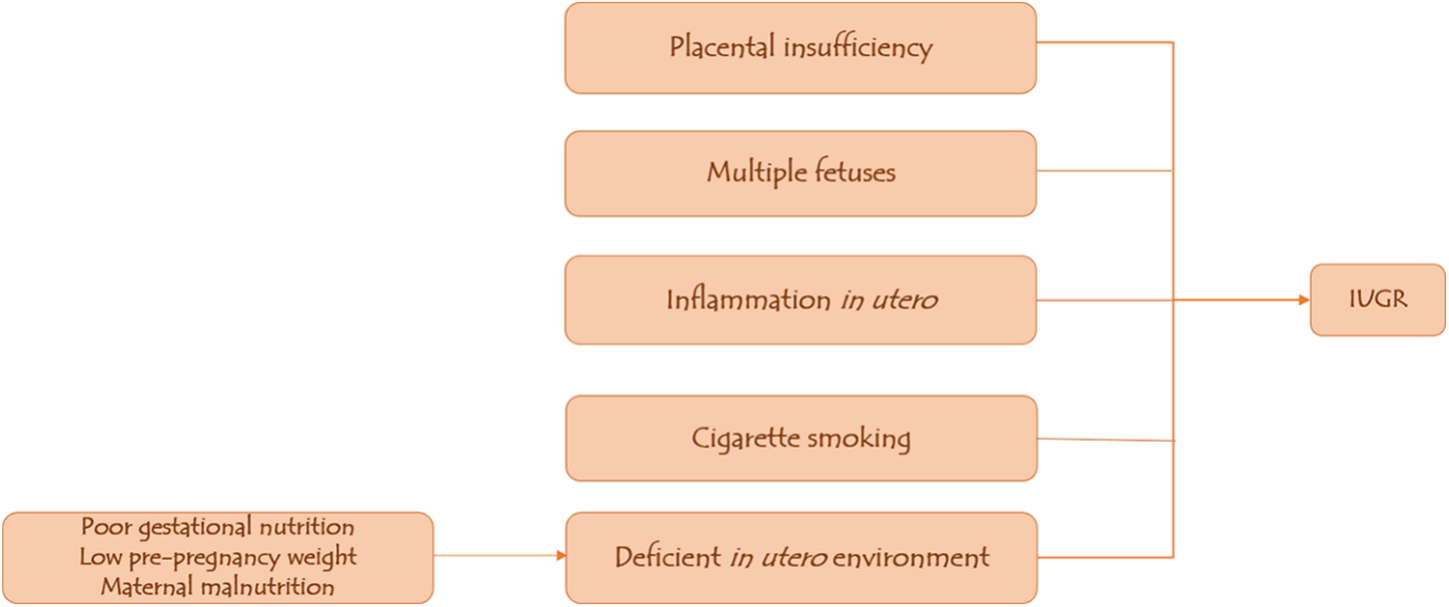
Predisposing factors to IUGR.

Multiple fetuses from twin pregnancies or assisted conception may result in nutrient competition between the two fetuses, predisposing them to IUGR; adolescent mothers are also at risk of having IUGR babies because their bodies compete for nutrients with the fetus (11, 17). Additional potential causes of intrauterine growth restriction (IUGR) include *in utero* inflammation, maternal malnutrition as low food intake and starvation result in a reduced nutrient stream from the mother to the fetus, thus restricting fetal growth; and placental insufficiency caused by fetal–placental perfusion dysfunction, which results in hypoxia and acidosis in the fetal circulation (18–20) (Figure 1).

## 3. Effects of IUGR on different organs

When exposed to *in utero* undernourishment, the available nutrients tends to be diverted to the growth and functional preservation of vital organs, e.g., brain, at the expense of organs such as the liver and pancreas (21). Even a slight reduction in nutrient supply limits fetal glycogen and fat formation, muscle and bone growth, with redistribution of cardiac output favoring delivery to the brain (2). The effects of environmental challenges are determined by their severity, duration, gestational age and probably the fetus's gender (22). The liver, the principal organ involved in the metabolism of dietary nutrients becomes a major target undergoing epigenetic, structural, and functional changes due to early exposure to unfavorable environment (23). The liver is essential in regulating gene expression for key transcription factors (24). Piglets with IUGR suffer from the hepatic disorder of lipid catabolism (25), with a significantly upregulated hepatic inflammatory factor under mRNA expression (26). It has been documented that IUGR piglets are presented with increased hepatic lipid concentrations, higher levels of serum pro-inflammatory cytokines, impaired growth, insulin resistance and inflammation (18).

Fatty hepatic infiltrates and cytoplasmic vacuolization has been reported in the livers of newborn IUGR piglets (27). An examination of the hepatic proteome revealed that the IUGR fetus had lower lipoprotein lipase activity (LPL) (28). IUGR piglets, compared to the normal birth weight (NBW) piglets, displayed low liver weight and compromised growth performance, decreased plasma free fatty acid (FFA) level, elevated hepatic oxidative stress (OS), abnormal hepatic lipid accumulation and compromised hepatic immune function. Some genes such as heme oxygenase 1, stearoyl-CoA desaturase 1, liver fatty acid- binding proteins 1, superoxide dismutase 1, toll-like receptor 4, sterol regulatory element-binding protein 1c, and tumor necrosis factor-alpha (TNF-α) have abnormal transcriptional expression in the liver of IUGR models (29). Therefore, it is a major requirement to improve the hepatic lipid metabolism, redox and immune status in the IUGR piglets’ model as it will be instrumental in developing new strategies for IUGR infants to prevent or/and slow the progression of non-alcoholic fatty liver disease (NAFLD) (29).

Through these findings, it is observed that among major defects occurring due to IUGR, lipid metabolism is greatly affected, followed by the immune function and its correlated genetic expressions. Thus, a hostile milieu can greatly impact an organism's normal biological function and development. The changes undergone by the organ for proper adaptation to the environment can be long-lasting and irreversible.

## 4. Preclinical IUGR investigations using different models

Diverse animal models of IUGR have been employed to investigate the mechanisms of intrauterine adaptation and programming, including maternal stress, uteroplacental insufficiency, hypoxia, nutrient (protein) restriction and glucocorticoid treatment (30). As the genetic background in animal models can be controlled, it is possible to explore the effects of the environment on long-term health during gestation or early postnatal life (22).

In multifetal mammals, IUGR has been reported to have lasting and harmful impacts on neonatal birth weight, postnatal growth, development, and liver function (31, 32). Pigs have been widely researched for human health regulation and animal nutrition. They have been extensively used as an animal model for IUGR studies due to their biological similarity to humans (33). Studies have shown that due to nutritional deficiencies during gestation, IUGR piglets displayed the most severe naturally occurring conditions; they have a higher risk of IUGR than other animals (25, 34).

The sheep model is attractive in studying the mechanistic basis of fetal adaptations that occur during fetal hypoxia, hypoglycemia, and IUGR caused by poor placentation (3). Other strengths of the sheep model include the availability of a singleton fetus, duration of pregnancy, similarities with humans in regard to organ and body development regulatory pathways and homeostasis. Most importantly, just like in humans, prenatal malnutrition in sheep has an extended impact on the endocrine, cardiovascular, and postnatal homeostasis systems (35).

Rodents offer a significant advantage in terms of relatively short gestation and lifespan (22). Malnutrition during critical early life periods has been shown to affect subsequent development (36) and perinatal adverse challenges have led to long-term consequences. For example, in both males and female rats, maternal caloric restriction results in low-birth-weight offspring with accelerated neonatal growth, early vaginal opening and sexual dimorphism (37, 38). However, some findings indicate that regardless of postnatal diet, male growth-restricted rats are resilient to impaired glucose homeostasis while female growth-restricted rats are more vulnerable to metabolic dysfunction (39). In another study the results demonstrated an intrauterine growth retardation and hypercholesterolemia in male adult offspring rats following prenatal nicotine exposure (PNE) (40). These findings highlight the role of gender in the development of IUGR associated Mets.

In summary the availability of various animal models makes it possible to study IUGR associated Mets. However, these models have their own advantages and disadvantages, therefore they are selected based on the research interest, need and available resources. Understanding the characteristics of each model is highly useful for the development of preventive, diagnostic and therapeutic strategies.

## 5. Impacts of intrauterine growth restriction

### 5.1. Restricted growth and development

IUGR is one of the leading causes of perinatal morbidity, affecting approximately 7%–15% of pregnancies worldwide (41, 42). Low birth weight (LBW) can be passed down through two generations; a low-birth-weight mother is 2.8 times more likely to have a low-birth-weight baby (43). LBW and IUGR are linked to preconception anemia (44). A large number of epidemiological and animal studies have reported that LBW caused by IUGR is mainly associated with an elevated risk for the development of NAFLD in both children and adults (45–48). IUGR can present with a SGA accompanied by LBW, enhancing lipid accumulation and other metabolic abnormalities. Stunted growth and development affects approximately 7%–9% of newborns, and may be responsible for up to 50% of unexplained stillbirths (19). About 86% of children born with small gestational age (SGA) catch-up with normal developmental pace and reach normal adult height with the others end up as short adults (49). Metabolic aberrations have been noticed in adult IUGR rats exhibiting catch-up growth (50). Children with IUGR, especially if they achieve catch-up growth in childhood, as well as SGA subjects, are at a higher risk for long-term developmental consequences or developing diseases later in life such as short stature, metabolic syndrome, Type 2 diabetes, hypertension, dyslipidemia, insulin resistance, and cardiovascular disease (7, 51). An enormous economic loss has been recorded in pig production due to the decreased survival rate of low-birth-weight piglets induced by IUGR (52).

### 5.2. Altered hepatic redox status

Oxidative stress is implicated in the IUGR-induced liver injury (53). Oxidative stress, particularly in the pancreas may be a common mechanism by which an adverse intrauterine milieu influences the development of the fetus and subsequent development of type 2 diabetes (54). An increase in the concentration of acidic metabolites (g-glutamyl leucine and 2 hydroxybutyric acids) may be associated with oxidative stress which might result in insulin resistance in the child (55). A recent study by Cheng et al. revealed increased plasma concentrations of malondialdehyde (MDA) and protein carbonyl (PC), and decreased total superoxide dismutase (T-SOD) in IUGR pigs (*longissimus lumborum*) compared with NBW pigs (52).

### 5.3. Genetic implications

The epigenetic adaptation of the fetus during the gestational period has lasting impacts on its growth and development. Maternal genes play a very important role in offspring development. For instance, pleckstrin homology-like domain-family A member (PHLDA2) regulates placental growth, growth receptor binding protein (GRB10) is associated with insulin receptor signal transmission, the IGF1 receptor inhibits the insulin receptor signal and placental acid-labile subunit (ALS) (56, 57). It has been reported that pigs suffering from IUGR displayed marked upregulation of mRNA expression for sterol regulatory element-binding protein-1 (SREBP-1), liver-x receptor α (LXRα), and peroxisome proliferator-activated receptor α (PPARα) (25). In mice, increased expression of CoA desaturase 1 (SCD1) gene, and decreased expression of carnitine palmitoyl transferase 1 alpha (CPT1α), acyl-CoA oxidase 1 (ACOX1), IGF-1 and IGF-2 genes have been reported (58).

Insulin and fatty acid regulation by lipogenic gene expression are largely mediated by transcription factors (such as SREBPs) and to a lesser extent, by nuclear receptors (such as LXRs) (59, 60). In mice, the insulin-like growth factor 2(IGF-2) gene is regulated by the maternal H19 gene, encodes IGF-2 synthesis and contributes to fetal growth. In contrast, the IGF2R gene, which encodes the IGF2R receptor, may cause intrauterine growth restriction (61).

### 5.4. Metabolic syndrome induced by IUGR

#### 5.4.1. Insulin resistance and its genetic correlation

The concentration of insulin in the serum is increased significantly due to IUGR, leading to low serum glucose concentration and a significantly decreased glycogen concentration in the liver (25). Children exposed to IUGR have increased insulin resistance, and low birth weight has also been linked to altered insulin sensitivity (62, 63). Insulin sensitivity is commonly defined as insulin's ability to stimulate glucose uptake in peripheral target tissues (64). Insulin resistance is regarded as the damage to insulin signal transduction occurring when normal hormone concentrations in the blood are insufficient to regulate metabolic pathways (25). In white adipose tissue, insulin increases fatty acid flux to the liver, resulting in ectopic fat deposition in hepatocytes (65). The increased visceral fat deposition eventually lead to insulin resistance in patients with growth hormone deficiency (GHD) (66). Insulin resistance can develop as early as one year of age in SGA children (67). Young men born with IUGR have 30% lower insulin secretion in comparison to their insulin sensitivity, indicating a lower insulin deposition index (68). Additionally, when postnatal nutrient availability exceeds prenatal predictions, there is increased postnatal growth and fat deposition resulting in insulin resistance (69). The “survival” hypothesis claims that peripheral insulin resistance occurs in order to redistribute glucose to vital organs (e.g., the brain) of a malnourished fetus (70).

Insulin receptor dysfunction caused by abnormal phosphorylation of the β-receptor subunit's tyrosine kinase also leads to insulin resistance (71). IGF-1 levels are related to beta cell function and growth in the first year, and at age three, IGF-1 is related to BMI and insulin resistance (72). Studies have shown that insulin resistance in a child with IUGR is linked to higher IGF-1 and BMI levels during postnatal catch-up growth (14). Insulin resistance is also common in people with severe IGF-1 deficiency (73).

#### 5.4.2. Type 2 diabetes mellitus and its genetic correlation

Infants born with severe IUGR and weighing less than 1.5 kg have lower beta cell mass resulting in Type 2 diabetes (74). Animals with impaired beta cell activity and low islet mass develop gestational diabetes, primarily caused by insulin deficiency (75). Fowden hypothesized that adult diseases, including diabetes, are caused, at least in part, by changes in the development of key endocrine axes, specifically the hypothalamic–pituitary–adrenal (HPA) axis, during suboptimal intrauterine conditions associated with impaired growth (76).

It has been demonstrated that intrauterine growth patterns can be linked to specific adult diseases; for example, a thin infant with a low ponderal index is more likely than a symmetrically small baby to develop type 2 diabetes as an adult (77). Low birth weight is associated with reduced expression of insulin signaling proteins in muscle and adipose tissue preceding the development of diabetes (22). In IUGR rats, altered hepatic glucose metabolism may contribute to the onset of fasting hyperglycemia prior to the development of obesity and diabetes (78). Fetal hyperglycemia is caused by severe maternal diabetes, puppies born to severely diabetic mothers with stunted intrauterine growth remain small until they reach adulthood (22).

Unfavorable intrauterine environment and the accumulation of DNA methylation errors over time may result in premature “epigenetic aging”, contributing to an increased susceptibility to diabetes in adulthood (79). Diabetes develops in IUGR rats with a phenotype similar to type 2 diabetes in humans, namely progressive dysfunction in insulin secretion and action (22). In adults with IUGR, Cyclin-dependent kinase inhibitor 1C (CDKN1C), a cell proliferation regulator that affects pancreatic β cells, can have a mutation at chromosome 11p15, resulting in decreased fetal growth, growth deficiency, and the onset of diabetes at a young age (80).

#### 5.4.3. Dyslipidemia

The key steps in intra-fetal lipid synthesis and lipid catabolism are regulated by total cholesterol (TC) and total triglycerides (TG) (81). Hepatic lipid accumulation is caused by an imbalance between lipid availability and lipid disposal, eventually leading to lipoperoxidative stress and hepatic injury (82). The disproportion of lipid metabolism plays a critical role in hepatic defect and injury in IUGR individuals (29). Lipoprotein lipase (LPL) catalyzes the triacylglycerol hydrolysis in circulating chylomicrons and very low-density lipoproteins (83). Hepatic lipase (HL) is essential for the hydrolysis of circulating triglycerides and phospholipids (28). Lipogenesis is increased in IUGR piglets (84). Some studies on lipid parameters showed that the concentration of free fatty acids (FFA), total cholesterol (TC) and total triglycerides (TG) was increased while a decrease was found in the activity of lipoprotein lipase (LPL), hepatic lipase (HL) and total lipase (TL) in IUGR piglets' liver (25, 29, 52).

Furthermore, adipocytokines have been linked to adult diseases associated with IUGR. Leptin plays a permissive role in pubertal development and reproductive function maintenance. Adiponectin has been shown to play a role in linking energy homeostasis and hypothalamo-pituitary-gonadal axis control. Ghrelin, an orexigenic compound, stimulates growth hormone secretion as well. PYY 3e36 is a gastrointestinal hormone that regulates food intake and energy balance; it has also been shown in animal studies to modulate GnRH and gonadotropin release (51).

Also, hyperinsulinemia is known to increase hepatic very-low-density lipoprotein synthesis, which may contribute to higher plasma triglyceride and LDL-c levels, and resistance to insulin action on lipoprotein lipase in peripheral tissues may also contribute to higher triglyceride and LDL-c levels [56]. Brown adipose tissue, which protects against metabolic disorders, was found abnormal in SGA children (85).

#### 5.4.4. Obesity

Obesity and overweight are linked to a variety of comorbid disorders, including cardiometabolic diseases (hypertension, diabetes, and insulin resistance), as well as some malignancies (86). The negative effects of “catch-up” growth in humans have been connected to the development of obesity (87). Insulin resistance in particular, is usually associated with obesity (88). Fetal malnutrition, pancreatic ß cell dysfunction, altered insulin metabolism, and, as a result, obesity are all associated to epigenetic regulation (89).

Obesity causes lower HDL cholesterol levels, higher systolic and diastolic blood pressures, higher triglyceride levels, and higher hemoglobin A1c (HbA1c) (90). Triglycerides are normally stored in adipocytes, but when energy intake exceeds the body's capacity, they can be stored in the liver, muscle cells, and visceral adipocytes (adipocytes surrounding vital organs), resulting in central obesity (91). Dams subjected to a much more severe food restriction of 30% of ad-lib intake results in IUGR offspring who develop hyperphagia and obesity as adults (92).

A cohort study showed that weight gain in the first three months of life was associated with more fat, central adiposity, lower insulin sensitivity, and higher insulin resistance in early adulthood, including in SGA children with catch-up growth (93). Also, low leptin levels, low or normal adiponectin levels, and higher ghrelin and visfatin levels in the IUGR sate may put an individual at risk for obesity development (94).

## 6. Recent and prospective IUGR therapies

Presently, IUGR has no known effective therapies (95). However, numerous treatments are being investigated to support the improvement of growth performance, immune status, lipid and glucose metabolism, prevention as well as treatment of obesity-related metabolic diseases and so on. In humans, therapies such as antenatal corticoids, aspirin, low molecular weight heparin, phosphodiesterase type 5 inhibitors, nitric oxide donors, N-acetylcysteine, proton pump inhibitors, and Maternal vascular endothelial growth factor (VEGF) gene therapy have been used to improve poor placentation and uterine blood flow (95, 96). Other drugs such as statins and melatonin were reported to have anti-inflammatory and antioxidant properties (96). Magnesium sulfate on the other hand has been widely used as prophylaxis for neuroprotection (95, 97). Similarly, in preclinical animal experiments several compounds are being investigated and could pave the way to future effective therapy. Table 1 summarizes some of these drugs.

**Table 1:** Intrauterine growth restriction preclinical experimentation.

Drugs (dosage)	Description	Observed Metabolic Therapeutic effects	Refs
Choline (180 mg/kg)	A precursor of PC involved in the formation of VLDL in the liver	Improved growth and decreased liver lipid in pigs. IUGR pigs require supplementary choline to improve PC production and maintain normal lipid metabolism.	(98)
Curcumin (400 mg/kg)	A phenolic compound derived from turmeric.	The dietary curcumin supplementation reduces subacute stress, improves the growth performance, intestinal mucosal barrier integrity, morphology, and immune status of pigs.	(18, 99)
DHA (0.25 mg/kg)	Antimalarial drug	Antibacterial, antitumorigenic, and antifibrotic properties. Prevention and treatment of obesity-related metabolic diseases.	(100–103)
GH	Primary regulator of postnatal growth.	Lipolytic effects, treats growth failure in SGA children who do not catch up until the age of two.	(100, 104, 105)
RSV (80 mg/kg body weight/d)	A natural polyphenol	Anti-oxidation, anti- inflammation, anti-cancer, lipid- lowering effect, anti-stress feed additive.	(29, 58–62, 63)
TB (0.1%)	A triglyceride	Attenuates insulin resistance and abnormal levels of lipid.	(25, 106)

PC, phosphatidylcholine; VLDL, very low-density lipoprotein; DHA, dihydroartemisinin; GH, growth hormone; RSV, resveratrol; TB, tributyrin.

## 7. Concluding remark

Although the data used are not exhaustive and conflicting, an in-depth understanding of the occurrence and development of metabolic disorders in IUGR models is key to promoting therapies that will ultimately tackle and mediate the consequences caused by these metabolic aberrations. Even though a clear knowledge of the underlined mechanism is still a challenge to overcome, the present studies are helpful in grasping the clinical impact of IUGR.
